# Silicon etching using only Oxygen at high temperature: An alternative approach to Si micro-machining on 150 mm Si wafers

**DOI:** 10.1038/srep17811

**Published:** 2015-12-04

**Authors:** Jessica Chai, Glenn Walker, Li Wang, David Massoubre, Say Hwa Tan, Kien Chaik, Leonie Hold, Alan Iacopi

**Affiliations:** 1 Queensland Micro and Nanotechnology Centre, Griffith University, Nathan, 4111 QLD, Australia

## Abstract

Using a combination of low-pressure oxygen and high temperatures, isotropic and anisotropic silicon (Si) etch rates can be controlled up to ten micron per minute. By varying the process conditions, we show that the vertical-to-lateral etch rate ratio can be controlled from 1:1 isotropic etch to 1.8:1 anisotropic. This simple Si etching technique combines the main respective advantages of both wet and dry Si etching techniques such as fast Si etch rate, stiction-free, and high etch rate uniformity across a wafer. In addition, this alternative O_2_-based Si etching technique has additional advantages not commonly associated with dry etchants such as avoiding the use of halogens and has no toxic by-products, which improves safety and simplifies waste disposal. Furthermore, this process also exhibits very high selectivity (>1000:1) with conventional hard masks such as silicon carbide, silicon dioxide and silicon nitride, enabling deep Si etching. In these initial studies, etch rates as high as 9.2 μm/min could be achieved at 1150 °C. Empirical estimation for the calculation of the etch rate as a function of the feature size and oxygen flow rate are presented and used as proof of concepts.

There are two main types of chemical-based Si wafer micromachining; isotropic and anisotropic silicon etching^1^,^2^. Isotropic etching is where the etch rates is identical for all crystal orientation, and is well-suited for processes such as structure release etch as well as to remove the sacrificial Si wafer after its use as a deposition template or mechanical support for device fabrication, is no longer needed^1^. Anisotropic etching is where the etch rates is dependent upon the crystal orientation of the etch direction. Anisotropic etching of Si is typically used when dimension control of the etching is critical, such as for formation of high aspect ratio features, deep Si trenches, or to exploit the anisotropy to precisely create specific patterns on wafers of particular crystal orientation, such as V grooves structures on Si(100) substrates^2^. Isotropic and anisotropic etching of Si have both wet and dry etching options, each with their own advantages and disadvantages. Wet etchants such as hydrofluoric:nitric:acetic acids (HNA) and potassium hydroxide (KOH) solutions are typically used for isotropic and anisotropic etching of Si, respectively^2^. While wet etchants are attractive due to the relatively simple setup requirements, they can suffer from drawbacks such as stiction, the handling of hazardous/toxic chemicals and expensive waste disposal management. Wet etching can also induce mechanical damage to the structures from liquid agitation and/or wafer drying processes that lead to yield and reliability variations^2^,^3^. The effect of stiction is particularly undesirable for the fabrication of micro-electromechanical systems (MEMS) as the thin structures can literally “stick” to the substrate, preventing safe release or operation.

On the other hand, common dry etchants such as xenon difluoride (XeF_2_), sulfur hexafluoride (SF_6_) alone or in combination with fluorocarbons (C_x_F_x_) (SF_6_ + C_x_F_x_), do not suffer from the detrimental stiction effect and can be easily calibrated and automated^2^,^4^. XeF_2_ is preferred over SF_6_ for isotropic etching of Si as XeF_2_ is a plasma-less process, whereas SF_6_ is a plasma process. A plasma-less process means that the etching gas is spontaneously reactive, whereas a plasma process requires external excitation to create reactive species, which requires additional costs to implement. By alternating C_x_F_x_ with SF_6_, the etching turns from isotropic to anisotropic Si etching as at each cycle one alternates between sidewall passivation (with C_x_F_x_) and trench etching with SF_6_ (ie. the so-called Bosch process)^5^. SF_6_ + C_x_F_x_ is currently preferred for anisotropic Si etching due to its ability to form extremely high aspect ratio trenches (with sidewall angle close to 90°)^2^, as there is currently no other alternative processes available that can achieve such characteristics. However, SF_6_ is a potent greenhouse gas^6^ and creates a toxic byproduct upon reaction with Si (ie. silicon tetrafluoride^7^). In addition, operating with dry etchants is usually more complex than wet etching and requires expensive equipment and the use of hazardous gas, which therefore requires appropriate waste abatement and disposal systems to be installed and maintained.

Ideally, the perfect Si etchant would be an etchant that does not cause additional damage to the wafer being processed, etch only in the pre-determined locations, has simple setup requirements, high etch rates and good uniformity over the entire wafer, high selectivity to common mask materials, uses easy-to-dispose chemicals, and does not create environmental-unfriendly/toxic by-products. With these objectives in mind, we present here an alternative Si micromachining method using only oxygen (O_2_) at high temperatures. This process meets many of the aforementioned properties as we will show in this paper.

Most studies of the reaction of oxygen with Si are primarily focused on the oxidation of Si for the purpose of converting the Si surface (after initially lightly etching the Si) into silicon dioxide (SiO_2_)^8^,^9^. Two possible outcomes may occur upon reaction of Si with O_2_: Si etching via reaction (1) 2Si + O_2(g)_ → 2SiO_(g)_, or Si oxidation via reaction (2) Si + O_2(g)_ → SiO_2(s)_. More details on this mechanism are available in a thorough study by Smith and Ghidini^10^. At high temperatures and low oxygen partial pressures, Si reacts with O_2_ to form the volatile gas silicon monoxide (SiO) (reaction (1))^8^. Conversely, at high temperatures and high partial pressures, solid silicon dioxide (SiO_2_) is formed on the exposed Si surface instead^8^ (reaction (2)). While both processes etch the Si substrate to some degree, only reaction (1) will enable continual etching of Si as reaction (2) is self-limiting since the SiO_2_ formation rate reduces over time due to increasing difficulty for O_2_ to diffuse through the thick SiO_2_. Therefore we have optimised process parameters to favour reaction (1), enabling deep Si etching and therefore achieve controllable Si micro-machining.

In this paper, O_2_ etching of Si was optimised for Si micro-machining. It is demonstrated that this Si etching technique provides fast and uniform etching of large Si wafers and is compatible with multi-wafer batch processing. Furthermore, it is shown that isotropic and anisotropic etching can be obtained based on process conditions. In addition, this process is inherently stiction-free (being a dry etchant) and is safer than toxic halogen based dry etchants, and so does not require expensive waste management systems. The main advantages and disadvantages of common Si etching techniques compared to our O_2_ based Si etching technique are summarised in Supplementary Table S1^1^,^2^,^5^,^11^.

Here, we report the initial results of using this alternative Si etching technique with three different hard masks: silicon carbide (SiC), SiO_2_, and silicon nitride (SiN) thin films. As proof of concepts, we will also demonstrate the fabrication of single crystal SiC-based diaphragms, of MEMS-like structures, and long Si stripes mimicking photonic waveguide structures. To the best of our knowledge, no such etching method (deep Si etching using only O_2_ at high temperatures for Si micro-machining) has been reported to date. Thanks to its many advantages, we believe that this alternative Si etching technique can become an attractive Si micro-machining process for a broad range of applications including MEMS and silicon photonics.

## Results and Discussion

By increasing the etch temperature from 1100 °C to 1200 °C at an O_2_ flow rate of 20 sccm, the etch rate increased by 63% (from 1.9 μm/min to 3.1 μm/min). Importantly, etching at 1150 °C gives a reasonable etch rate (2.7 μm/min) and implies that a custom-built O_2_ etching equipment can use electronic grade quartz (maximum temperature ~1160 °C) in its construction. Hence 1150 °C is the etch temperature used for subsequent studies.

**Figure 1:**
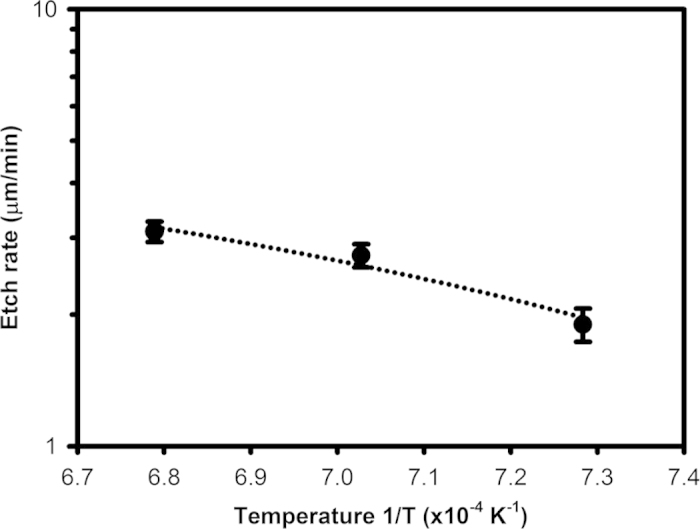
Arrhenius plot showing the etch rate measured on a 600 μm wide square after etching with 20 sccm O_2_ at 1100 °C to 1200 °C. The dash line indicates a linear least squares fit, with an adjusted coefficient of determination R^2^ of 0.87.

An Arrhenius plot displayed on Fig. 1 and plotting the etch rate versus inverse temperature (1/T) yields an activation energy of 0.86 eV for O_2_ etching, higher than the activation energy of Si(100) etching using XeF_2_ (0.26 eV above 410 K)^4^. A higher activation energy with O_2_ etching is expected in this process since the O_2_ molecules are converted into reactive O species at high temperatures^10^, whereas XeF_2_ is highly reactive at much lower temperatures^2^. The higher temperatures required for O_2_ etching of Si is therefore consistent with the higher activation energy. The etching behaviours for subsequent sections are examined in terms of etch rate, anisotropy, uniformity, and mask selectivity.

**Figure 2:**
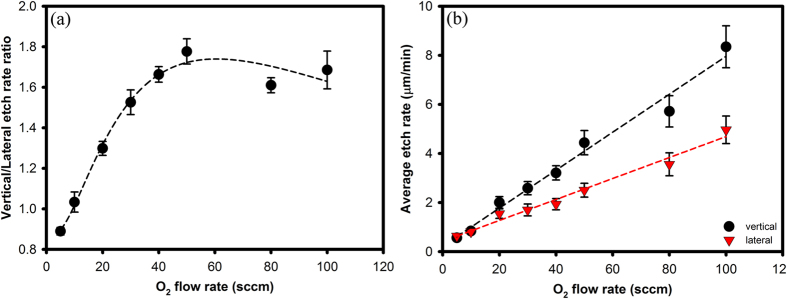
(**a**) Ratio between the vertical and lateral etch rates as a function of O_2_ flow rates. The dashed line is a line guide to the eye. (**b**) Average vertical and lateral etch rates as a function of O_2_ flow rates. The error bars both (**a**,**b**) indicate the range of etch rates measured for all the different square opening widths at a given O_2_ flow rate. Dashed lines indicate least squares fit to a linear function for the plot in (**b**). All experiments were performed at 1150 °C with epitaxial SiC masks.

Firstly, we examine the effect of O_2_ flow rate on the Si etch rate. Due to the high aspect ratio of the test patterns, the vertical and lateral etch rates at various O_2_ flow rate were measured and compared using a combination of optical microscopy (OM) and scanning electron microscopy (SEM) imaging. Lateral and vertical etch rate results at various O_2_ flow rates are plotted on Fig. 2. They were found to be fairly similar for a flow rate of 10 sccm O_2_ at 1150 °C (ie. isotropic etching), but anisotropic outside 10 sccm O_2_, as shown in Fig. 2(a). However, Fig. 2(b) shows that both the vertical and lateral etch rates increase at a quasi-linear rate with O_2_ flow rate and so the saturation in the vertical-to-lateral etch rate ratio is not due to a vertical etch rate saturation at high O_2_ flow rates (>50 sccm).

The anisotropic etching behaviours at different O_2_ flow rates is currently under investigation but might be related to various competing mechanisms such as chemisorption, surface diffusion, transition from molecular to viscous flow, and changes to O_2_ sticking coefficients. A detailed study will be reported in another manuscript. Similar anisotropic behaviour has also been observed in XeF_2_ etching, where the degree of anisotropic was found to be dependent on exposed Si area, XeF_2_ charge pressure, and number of etch cycles^12^. However, due to the approximately 3–4 orders of magnitudes pressure difference for XeF_2_ operation (~4 Torr operation pressure) compared to this O_2_ etching (10^−3^ to 10^−4^ Torr operation pressure), the anisotropy mechanism cannot be identical. For example, etch rate anisotropy in XeF_2_ is shown to be primarily caused by a short mean-free-path of ~20 μm^13^, but the mean-free-path during O_2_ etching operation is in the centimetre to metre range.

Nevertheless, Fig. 2(b) show that the general trend is that higher O_2_ flow rates leads to higher etch rates. The higher etch rate at higher O_2_ flow rates is expected as more O atoms are available for etching. The vertical etch rates achieved varied between ~0.7 to 9.2 μm/min for O_2_ flow rates from 5 to 100 sccm at 1150 °C, much faster than the typical reported wafer-level etch rates^12^ of 0.2–0.5 μm/min with XeF_2_ and is comparable with typical HNA and KOH^14^ etch rates (microns per minute). The 9.2 μm/min etch rate with O_2_ etching at 100 sccm O_2_ is also comparable with high etch rate, microwave enhanced KOH etching of ~10 μm/min^14^. It is worthwhile noting that etch rate is limited here by our equipment and that higher etch rate is expected for higher O_2_ flow rate, i.e higher O_2_ pressure. This illustrates that O_2_ etching combines advantage of fast etch rates of HNA with the stiction-free etching of dry etchants. Furthermore, an important issue with XeF_2_ etching is that the etch rate varies over the etching time. One cause of this is due to self-heating of the substrate during etching, which result in decreasing etch rates as etch time increases for etch temperatures below 400 K^4^,^15^. Another cause is due to increasing difficulty for XeF_2_ to diffuse into a cavity as its depth increases^12^. To evaluate this possible issue with the O_2_ etching, we compared two etching runs done at 1150 °C using 10 sccm O_2_ with etch times of 30 mins and 75 mins respectively and found that the etch rates were identical. This implies that O_2_ etching does not suffer from time-dependent etch rate changes as typically seen in XeF_2_ etching.

**Figure 3:**
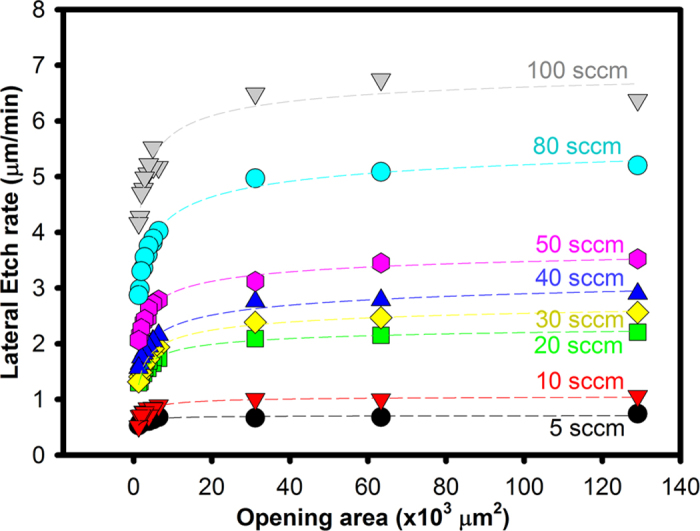
Effect of the mask opening area on the lateral etch rate for Si(100) at different O_2_ flow rates. The etch temperature was 1150 °C. The dashed lines represent a fit to an empirical power law function. The measurement error is at most 5%, based on the resolution of the optical microscope.

The variations in lateral etch rates as a function of mask opening area is plotted in Fig. 3. It was found that the experimental etch rates are well fitted by an empirical power law function given in Eq. (1), where ER is the lateral etch rate in μm/min, area is the mask opening area in μm^2^, and a,b, and c are fitting variables that depend on the O_2_ flow rate (or partial pressure) (sccm) and etch tool design. The adjusted coefficient of determination R^2^ values are calculated to range between 0.89 and 0.99 for all power law function fits (see Fig. 3). The vertical etch rate can be deduced from the vertical-to-lateral etch rate ratio shown in Fig. 2(a).


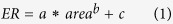


The etch rate variation is more pronounced for smaller feature areas, especially at high O_2_ flow rates, whereas the etch rate is mostly constant for larger features (>30 × 10^3^ μm^2^). Such area dependent etch rate has also been observed with XeF_2_ etch process, and is linked to the *so-called* microloading effect^16^. This effect occurs due to the diffusion-limited nature of etching, and hence a small mask aperture results in less volume of reactants able to diffuse through the opening and etch the underlying Si substrate. In addition, the etch rate variation for different feature sizes is minimal at lower O_2_ flow rates (<10 sccm) or for large features (>30 × 10^3^ μm^2^) at any O_2_ flow rate. Similarly, Sugano *et al.*^17^. have previously reported that a lower supply of XeF_2_ resulted in reduced etch rate variations with changes in feature size. Xu *et al.*^12^. have also reported that size dependent etch rate is minimised for large features, similar to that observed in Fig. 3. Appropriate design rules, similar to those created to handle the microloading effect in reactive ion etching (RIE)^18^ and XeF_2_ etching^12^, can be developed to overcome or even exploit this microloading effect, as in the case of RIE^18^. The etching behaviour as fitted by Eq. (1) can be used to assist in creating appropriate design rules.

**Figure 4:**
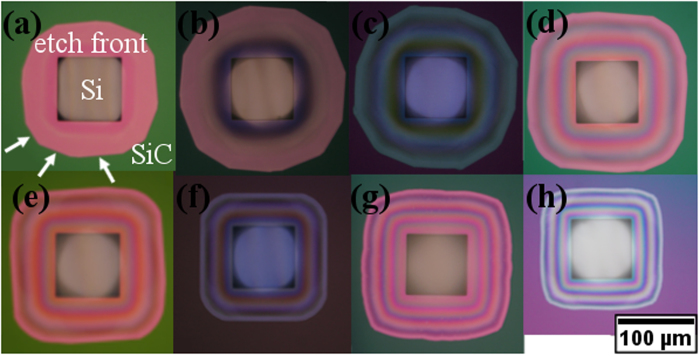
Optical microscopy images of 80 μm square mask openings after etching at 1150 °C under O_2_ flow rates of (**a**) 5, (**b**) 10, (**c**) 20, (**d**) 30, (**e**) 40, (**f**) 50, (**g**) 80, and (**h**) 100 sccm. The mask is SiC and the substrate is Si(100). The etch time varies between 7.5 and 75 mins. Scale bar is shared. White arrows indicate examples of facets. The etch depth depends on the O_2_ flow rate and is given by Fig. 2. The etch depth for these features range from 40 μm to 94 μm deep.

In addition to the observation of O_2_ flow rates dependent microloading effects, we have also observed lateral etch rate anisotropy depending on the O_2_ flow rate. Figure 4 shows that the etch front faceting also varies depending on the O_2_ flow rate. For O_2_ flow rates of 30 sccm and below the under-etch region appears as a multi-sided polygon, direct evidence of an anisotropic etching. At O_2_ flow rate of 40 sccm and above, the number of sides reduces such that it is mostly a square with rounded corner, which better mirrors the original square opening.

For a passivated Si surfaces, Si voids have been known to spontaneously form after annealing the Si at high temperatures^19^. The formation of voids has been attributed to Si out-diffusion from the Si substrate due to Si vacancy diffusion and aggregating into larger Si voids^20^. The Si voids formed due to high temperatures are typically quite small and are at most tens of nm wide for every minute of annealing at 1100 °C^19^. In our O_2_ etching process, the void formation is controlled as the Si is only exposed in the mask openings and protected elsewhere by the mask. With the addition of O_2_, the Si void formation is accelerated through formation of volatile oxides (SiO) via reaction of O atoms with Si surface dangling bonds, in addition to the spontaneous void formation purely due to high temperatures, resulting in several orders of magnitudes difference in Si void formation, and hence higher Si etch rates.

**Figure 5:**
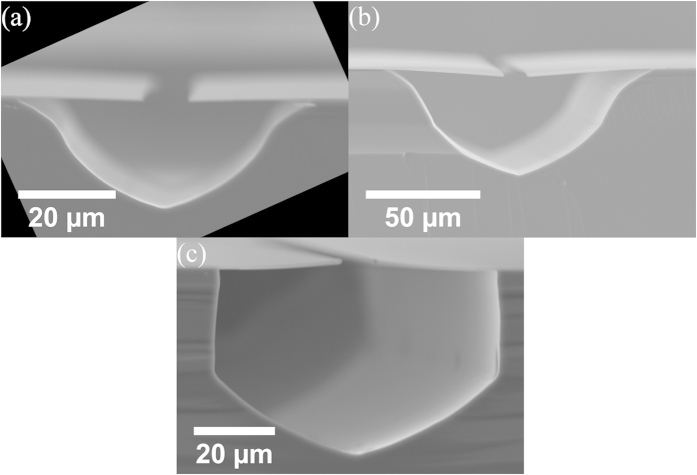
Cross-sectional SEM micrographs Si trenches formed from 10 μm wide mask openings after etching at 1150 °C under O_2_ flow rates of (**a**) 10 sccm for 30 mins, (**b**) 10 sccm for 75 mins, and (**c**) 100 sccm for 7.5 mins. The mask is SiC and the substrate is Si(100).

Figure 5 shows examples of cross-sectional SEM micrographs of Si that have been etched with 10 and 100 sccm O_2_ at 1150 °C, with a mask opening of 10 μm wide. The shape of the etched Si depends on the O_2_ flow rate, consistent with observation of lateral etch rate anisotropy in Fig. 4. The similarity in etched Si profile between Figure 5(a,b) confirm that that the etch profile depends on the O_2_ flow rate, as the specific profile only expands, and not change shape, with a longer etch time. The appearance of several intermediate high indices, fast etching planes prior to being eventually bounded by the slowest etching planes has previously been observed in KOH etching of Si^21^. The difference between etch rates of the different planes have also been found to be dependent on the KOH concentration^21^. As the lateral etch rate anisotropy gradually reduces at higher O_2_ flow rates, the mechanism for facet formation is likely related to the supply of O atoms and available Si bonds for etching, behaving in a similar manner to KOH etching. The detailed physical process involved in lateral etch anisotropy formation mechanism is currently under investigation and will be reported in a later manuscript.

However, in applications such as structure release and through-Si etch applications, faceting is not an issue. The faceting is also negligible when comparing it with the large features (channels may be centimetres long) that are typically used for microfluidic applications.

**Figure 6:**
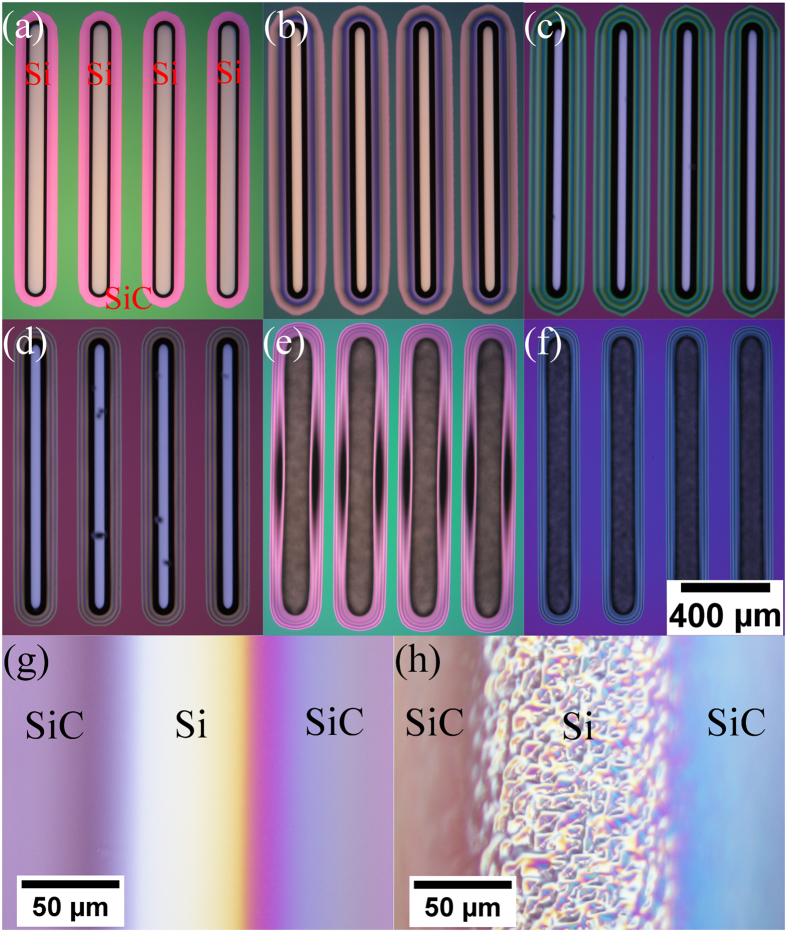
Optical microscopy images of 100 μm wide openings, 200 μm apart, and 1 mm long, after etching in (**a**) 5, (**b**) 10, (**c**) 20, (**d**) 50, (**e**) 80, and (**f**) 100 sccm O_2_ at 1150 °C. Higher magnification, Normaski images of the Si trenches etched with (**g**) 10 and (**h**) 100 sccm O_2_ at 1150 °C. The mask is SiC and the substrate is Si(100). The etching time varies between 7.5 mins to 75 mins. Total Si lateral etch is similar for all O_2_ flow rates. Scale bar is shared for (**a**) to (**f**). The etch depth depends on the O_2_ flow rate and is given by Fig. 2. The etch depth for these features range from 48 μm to 84 μm deep. No clear SiC mask edges are observable in (**g**) and (**h**) due the difference in height between the bottom of the etched Si trench and the top of the SiC mask.

We have also observed that the O_2_ flow rate affects the etched Si surface roughness. Smooth etched Si surfaces appear as a bright, white colour, and appear featureless under OM (cf. Fig. 6(g)). Figure 6 shows that from 80 sccm O_2,_ the etched Si is rough and can be observed under OM, through appearance of grainy black coloured trenches due to light scattering from the rough surface (cf. Fig. 6(h)). Kosolobov *et al.*^9^ and Ross *et al.*^22^ have previously reported that low pressure oxygen etching of Si at high temperatures proceed in a step-flow mode, analogous to high quality thin film epitaxy. The roughness at high O_2_ flow rates may arise from insufficient surface atom diffusion, similar to the situation that occurs in thin film depositions where surface atoms diffusion is limited by high adatom arrival rates. Essentially at low O_2_ flow rates, the Si is removed monolayer-by-monolayer, preserving the smooth Si surface. However at high O_2_ flow rates, Si is unevenly removed and results in rough etched Si surfaces.

**Figure 7:**
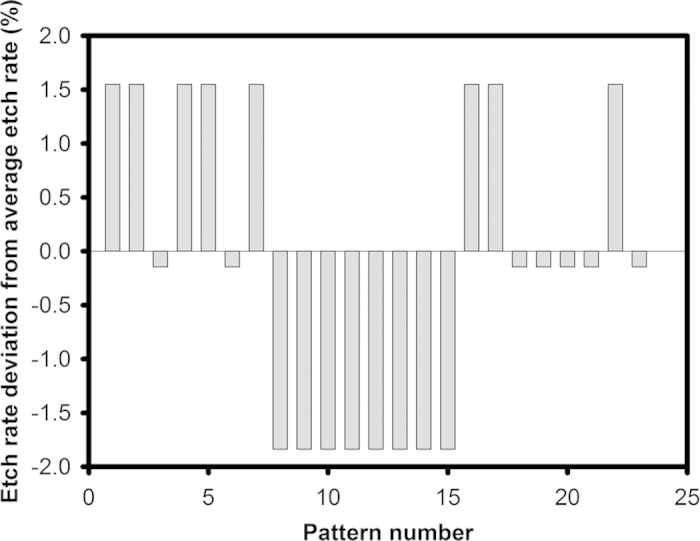
Etch rate deviation of multiple 100 μm wide, 1 mm long apertures spaced 4.5 mm apart across a 150 mm Si wafers etched with an O_2_ flow rate of 10 sccm at 1150 °C for 30 mins. The average etch rate is 1.23 μm/min.

Another important characteristic of an etchant is its cross wafer etch uniformity, as high yield is important for large-scale manufacture. A patterned SiC mask with multiple duplicates of 100 μm wide, 1 mm long apertures spaced 4.5 mm apart across a 150 mm Si wafer, resulting in ~40% exposed Si area, was used to investigate the etch uniformity across the wafer for the etch parameter of O_2_ flow rate of 10 sccm at 1150 °C for 30 minutes. The etch rate was found to be highly uniform across the wafer for a given aperture size, with an average etch rate non-uniformity of 4% (Fig. 7). Furthermore, a two-inch wafer patterned with the same features (~23% exposed Si area) was also loaded during the same etching run. The etch rates between the 50.8 mm and 150 mm wafer had a difference of only 4%. It is estimated that approximately 10% of O_2_ atoms are consumed when etching 23% of a two-inch wafer using an O_2_ flow rate of 10 sccm, which is consistent with observation of minimal loading effects with multi-wafer runs. The consistent etch rate across the wafer and for multi-wafers is important for process control in manufacturing to enable high device yield.

Following on the theme of manufacturability, another important characteristic is mask selectivity. Mask materials consisting of SiO_2_, SiN, and SiC were heated at 1150 °C under several different O_2_ flow rates and etch times. For all the tests, no mask degradation was observed under optical microscope. The selectivity is estimated to be greater than 1000:1 for all the samples which is more than sufficient for deep Si etch applications. This demonstrates that O_2_ etching is compatible with the common hard mask materials used for dry etching.

**Figure 8:**
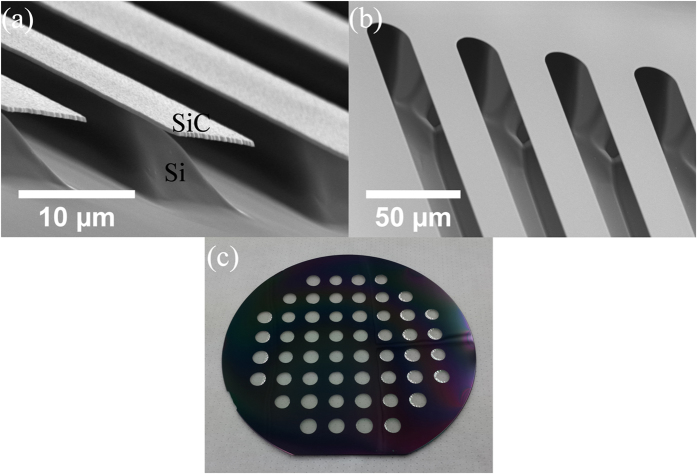
(**a**) Scanning electron microscopy image of SiC cantilevers after etching underlying Si with 5 sccm O_2_ for 15 mins at 1150 °C (Etch depth ~ 9 μm). (**b**) Scanning electron microscopy image of suspended SiC bridges on Si. The SiC bridges are 25 μm wide and 1 mm (Etch depth ~ 44 μm). (**c**) Multiple 8 mm diameter etch-through openings evenly spaced across a 150 mm Si wafer that have been coated with 500 nm SiC. This wafer has been etched at 1150 °C for 4 hours at 50 sccm O_2_. The Si wafer thickness is 675 μm. All transparent SiC windows indicate areas where the Si has been completely etched away, leaving just the 500 nm SiC thin film.

To demonstrate the capabilities of this new Si etching technique, we fabricated several building blocks typically found in Si-based applications such as MEMS and Si photonics (see Fig. 8). Firstly, we fabricated simple SiC cantilevers on a Si substrate (see Fig. 8(a)), which is a typical building block for MEMS^2^. The scanning electron microscope in Fig. 8(a) shows that the etched area is relatively smooth after 15 min etching using 5 sccm O_2_, unlike those typically seen after XeF_2_ etching, where the roughness is clearly visible under SEM^3^,^12^,^17^. The SiC cantilevers are also intact with no observable feature deformations. Next, we demonstrate fabrication of SiC bridges on a Si substrate, which mimics suspended SiC optical waveguides^11^. The bridges (Fig. 8(b)) show no obvious deformations and a smooth Si surface, indicating suitability as a building block for SiC-based Si photonics devices. Subsequently, we demonstrate formation of SiC diaphragms, which can be used as pressure sensor^23^. Figure 8(c) shows an array of 500 nm-thick SiC diaphragms that are ~8 mm in diameter formed from etching through the Si substrate from the backside. All SiC diaphragms survive the O_2_ etching process, leaving transparent SiC windows on the Si substrate. These examples show and ascertain the potential that O_2_ etching is suitable for creating a variety of structures for a broad range of applications.

## Conclusion

A novel technique for deep etching of Si using pure O_2_ at high temperature is introduced and used to successfully demonstrate a variety of micron-scale structures. Effects of the etch temperature, O_2_ flow rate, and mask aperture were investigated. Higher temperatures and higher O_2_ flow rates result in higher Si etch rates, although smooth etched Si surfaces is achieved only for O_2_ flow rates below 50 sccm. Vertical-to-lateral etch rate ratio varies depending on the O_2_ flow rate, with it being completely isotropic at 10 sccm O_2_. An interesting observation was that the lateral etch rate anisotropy can be controlled from anisotropic to isotropic by varying the O_2_ flow rate, with the transition to isotropic occurring at 30 sccm. High etch rate and high quality (smooth) etched Si surface coupled with the elimination of halogens (improves safety) and eliminating chemical waste hazards achieved in this initial study shows great potential for its use in large scale manufacturing.

## Methods

### Preparation of test wafers

2 inch and 150 mm Si(100) substrates were first coated on both sides with a thin film for etch mask purpose and consist of either ~300 nm thick single-crystal SiC using a customised low-pressure chemical vapour deposition (LP-CVD) tool^24^, ~100 nm thick SiN by reactive sputtering, or 100 to 1000 nm thick SiO_2_ formed either by using LP-CVD from SiH_4_ and O_2_ at 440 °C or thermally grown in a furnace by dry oxidation at 1000 °C. These three hard masks were tested for compatibility for this Si etching process but only the SiC mask was used for these studies for convenience as we had many SiC coated Si wafers already fabricated. Photoresist was then spin-coated onto the mask layers and then patterned using standard UV photolithography techniques, followed by dry etching to transfer the features of various shapes and dimensions between 2 to 600 μm wide and up to 1 mm long into the hard mask. The two inch wafers had an exposed Si area of approximately 23% and the 150 mm wafers had between 15% and 40% exposed Si areas.

### O_2_ etching of test wafers

Si etching was performed using the same LP-CVD system used for the SiC thin film deposition^24^. Due to the batch reactor nature of this LP-CVD system, between two to four two-inch patterned wafers can be loaded at the same time. The samples were heated up to either 1100 °C, 1150 °C or 1200 °C and then exposed to oxygen flows varying from 5 to 100 sccm (maximum flow possible) and for different etch times, transferring the various patterns into the Si substrates. After etching, the samples were cooled down to room-temperature in vacuum.

### Etched wafers characterisations

The lateral and vertical etch rate of the O_2_ etched samples were measured using an Olympus MX50 optical microscope (OM), a Jeol JSM-6510LV scanning electron microscope (SEM), and a Dektak 150 profilometer. The etch depth was determined using OM, SEM or a Dektak profilometer. Although OM measurements correlated to Dektak measurements for shallow etch depths, it should be noted that Dektak measurements were found to be unreliable for the narrower opening widths due to the difficulty for the relatively large stylus to reach the bottom of trenches which can be up to 100 μm deep. Hence OM was the predominant technique used to measure the etch depth. The lateral etch front anisotropy is studied using the OM. The etched Si roughness was evaluated using the OM and SEM. The mask selectivity was determined by measuring the mask thickness change before and after O_2_ etching using a Nanometrics Nanospec AFT 210 reflectometer.

## Additional Information

**How to cite this article**: Chai, J. *et al.* Silicon etching using only Oxygen at high temperature: An alternative approach to Si micro-machining on 150 mm Si wafers. *Sci. Rep.* **5**, 17811; doi: 10.1038/srep17811 (2015).

## Supplementary Material

Supplementary Information
