# Supplementary material for: Silicon etching using only Oxygen at high temperature: An alternative approach to Si micro-machining on 150 mm Si wafers
Source: Sci Rep. 2015 Dec 4;5:17811. doi: 10.1038/srep17811 (PMC4669475; doi:10.1038/srep17811)
Supplement: Supplementary Information [file srep17811-s1.pdf]

# Supplementary Information

## Silicon etching using only Oxygen at high temperature: An alternative approach to Si micro-machining on 150 mm Si wafers

J. Chai[1,]*, Glenn Walker[1], Li Wang[1], David Massoubre[1], Say Hwa Tan[1], Kien Chaik[1], Leonie Hold[1], Alan Iacopi[1]

[1]Queensland Micro and Nanotechnology Centre, Griffith University, Nathan, 4111 QLD, Australia.

*corresponding author: jchai@ieee.org

**This file includes:**

Supplementary Table S1

**Supplementary Table S1: Summary of main advantages and disadvantages of common Si etching techniques compared with $O_2$ based Si etching.**

| Etch properties | Wet etchants | | Dry etchants | | |
|---|---|---|---|---|---|
| | **KOH** | **HNA** | **$SF_6$ + $C_xF_x$** | **$XeF_2$** | **$O_2$ process** |
| Anisotropic etchant | √ | X | √ | X | $O_2$ flow rate dependent |
| Isotropic etchant | X | √ | X | √ | |
| Etch rates in several microns per min | √ | √ | √ | X | √ |
| Simple equipment requirements | √ (unless µwave enhanced) | √ | X | X | X |
| >100:1 selectivity to $SiO_2$ or $Si_3N_4$ mask | √ | √ | X | √ | √ |
| Hazardous chemicals | √ | √ | √ | √ | Not at the flows used |
| Hazardous/toxic waste disposal | √ | √ | √ (Very high global warming potential) | √ (Very high global warming potential) | No toxic by-product |
| <100° C low temperature etching | √ | √ | √ | √ | X |
| Stiction-free | X | X | √ | √ | √ |
| Regular liquid replenishment and stirring necessary to maintain etch rate | √ | √ | X | X | X |
| Multi-wafer process | √ | √ | X | X | √ |
